# Positive School and Classroom Environment: Precursors of Successful Implementation of Positive Youth Development Programs

**DOI:** 10.1100/tsw.2008.126

**Published:** 2008-10-10

**Authors:** Rachel C. F. Sun, Daniel T. L. Shek, Andrew M. H. Siu

**Affiliations:** ^1^ Social Welfare Practice and Research Centre, The Chinese University of Hong Kong, Hong Kong; ^2^ Quality of Life Centre, Hong Kong Institute of Asia-Pacific Studies, The Chinese University of Hong Kong, Hong Kong; ^3^ Kiang Wu Nursing College of Macau, Macau; ^4^ Department of Rehabilitation Sciences, The Hong Kong Polytechnic University, Hong Kong

**Keywords:** positive youth development, Project P.A.T.H.S., positive school culture, school related factors

## Abstract

This case study was based on a school where the Tier 1 Program of the Project P.A.T.H.S. was integrated into the formal curriculum. In this case study, an interview with the school principal, vice-principal, and social worker was conducted in order to understand their perceptions of administrative arrangements and issues in the school, implementation characteristics, program effectiveness, program success, and overall impression. Results showed that several positive school and classroom attributes were conducive to program success, including positive school culture and belief in students' potentials, an inviting school environment, an encouraging classroom environment, high involvement of school administrative personnel, and systematic program arrangement.

### INTRODUCTION

The Tier 1 Program of the Project P.A.T.H.S. (**P**ositive **A**dolescent **T**raining through **H**olistic **S**ocial Programmes) is a positive youth development program designed to promote holistic development of the junior secondary school students in Hong Kong using a curricular approach. Although the effectiveness of the program has been documented by research studies that use various evaluation strategies and data sources[1,2,3,4,5], few attempts have been taken to examine the school variables that are conducive to positive outcomes of such a newly adopted program in schools. As such, there is a need to delineate prominent school administrative and implementation characteristics that influence the process and quality of program implementation in the framework of school ecology.

A review of the literature showed that several macroschool environment factors are related to successful program implementation. These include a suitable physical environment, such as being physically safe and provision of sufficient space, facilities, and equipment for program implementation[6]; having nonauthoritarian and positive working environment, where staff can openly communicate and collaborate with each other, to enable staff's morale and passion for serving the youths[7]; and having positive ethos and norms, and creating a psychologically secure and supportive environment where there are abundant opportunities for the students' development[6]. Studies also showed that an inviting school environment where students feel they are a part of the school, are valued and accepted by their teachers and classmates, and can earn a valued identity was essential in strengthening the students' learning and positive development[8,9,10,11,12,13].

To create and maintain an inviting school environment that is conducive to program implementation, the leadership and support of the school principal was particularly indispensable. In Hong Kong school contexts, there is a division of labor between the school principal and vice-principal. The former is responsible for handling external administrative duties and making decisions that govern the school, while the latter is responsible for coordinating internal administration and teaching. Both of them are important in helping program administration and execution. At the program administration aspect, research studies showed that the principal plays a critical role by agreeing to the adoption of the program in the school, adoption of the program rationales as parts of the school ethos, incorporation of the program into the school formal curriculum, and setting clear goals for running the program[14,15,16,17]. At the program execution aspect, high involvement of the school principal in the program implementation creates a role model that encourages staff devotion and student involvement, thus raising the program effectiveness. Hence, the involvement of school administrative personnel is a key to unlocking program success.

The classroom environment is also an important element for program implementation. According to Trickett and Moos[18], a good classroom environment should (1) have good relationships, in which teachers support students, and students are highly committed and involved; (2) promote personal growth, in which there are clear goals for learning, and tasks and opportunities for independent development; and (3) possess system maintenance and allow changes, in which there are orderly organization and clear expectations, rules and regulations to maintain control, and innovations in teaching and learning.

To build a welcoming classroom environment that is suitable for implementing a positive youth development program, the teachers' agreement with the program philosophy and dedication is vital[14,19]. To maintain the program implementation quality, Weissberg[20] stressed that the teachers need to be trained, equipped with professional skills, possess a wide range of teaching skills, and be able to teach according to the students' interests and needs. Moreover, teachers should ensure that the teaching process is focused on the students' active participation and relationship building, consists of opportunities for practice and exercise, and comprises a caring, trustful, and committed learning atmosphere. Apart from these, several attributes have been put forward to increase program effectiveness at the program implementation level[14,21,22].

Finally, in order to sustain a new program in a school, incorporating the program into the school's formal curriculum with high program fidelity and a clear manpower deployment for program implementation is essential[21,23]. With particular reference to Hong Kong, as teachers are commonly overloaded with teaching and administrative tasks, and additional guidance duties that may lead to burnout[24], program integration would help to reduce the teachers' workload and thus their reluctance to implement a new program in the school[14]. In addition, a good manpower deployment plan and fair division of labor when launching the program would allow teachers to have more time to prepare for their roles and match them with those of others, and help to relieve teachers' burnout.

To examine how a macroschool environment (e.g., involvement of school principal and teachers) and microclassroom environment would promote the implementation quality of a positive youth development program in Hong Kong, a case study was adopted. In the specific context of Hong Kong, school principals have much authority in the school. Furthermore, classroom learning in Hong Kong stresses primarily on discipline and less on open expression of feelings and novelty. As such, it is important to understand how the classroom environment might contribute to the implementation of positive youth development programs in Hong Kong.

## METHODS

### Participants and Procedures

Among the 207 schools that joined the Project P.A.T.H.S. (Secondary 1 Program) in the Full Implementation Phase in the school year 2006/07, an exemplary school with good administration and implementation was invited to participate in this case study.

First, the school contact person was required to complete a questionnaire about the school's background information. Second, a 3.5-h focus group interview with the school principal, vice-principal (who was the contact person of the Project P.A.T.H.S.), and the school social worker was conducted by the third author and a trained colleague with a Bachelor degree in Social Work when the school had completed the Tier 1 Program. Prior to the study, consent from all informants was sought, and confidentiality as well as anonymity of the data collected were repeatedly emphasized.

The interview was conducted in Cantonese with reference to the semi-structured interview guide. There were several parts in the interview guide:

- Respondents' perceptions of the school administrative arrangement, such as preparation, support within the school, and from the social work agency
- Respondents' perceptions of the program implementation process
- Respondents' perceptions of program effectiveness, program success, and overall impression

### Data Analyses

The interview was tape recorded and the recordings of the interview were transcribed. The qualitative data were analyzed using general qualitative analyses techniques[25]. Preliminary data analyses were conducted by the trained colleague, and further checked by the first and second authors to ensure the reliability in the interpretation process. In addition, the authors addressed any personal biases and preoccupations that might influence the data interpretation.

## RESULTS

### School Characteristics

As shown in Table 1, the school adopted the 10-h core program of the Project P.A.T.H.S., which was incorporated into a school teaching subject (Moral Education). The school vice-principal, one teaching assistant, and one school social worker were responsible for delivering the program across four Secondary 1 classes (120 students in total). There was a clear division of labor, in which the vice-principal was responsible for monitoring classroom discipline, grouping students for activities, and sharing personal experiences, while the social worker was responsible for preparing teaching materials, coordinating the program-related matters, leading classroom discussion, implementing rewards to students, and following-up with the students' needs.

### School Administrative Arrangements and Related Issues

The school administrative arrangements and related issues are summarized in Table 2. Several observations could be highlighted. First, the school identification with the project rationales was essential to the program's implementation. The vice-principal pointed out that although their students were regarded as having low academic competence, he still had positive beliefs about the students' potential for

**TABLE 1**
**Summary of the Characteristics of the School**

| | |
|---|---|
| **I. Basic Information of the School** | |
| Total no. of Secondary 1 students (no. of Secondary 1 classes) | 120 (four classes) |
| Class teacher system | Double class teachers system |
| Participation in life education program organized by other organizations | Basic Life Skills Training Program, Project "I CAN" |
| No. of uniform/social and voluntary services groups in school | Four |
| Small groups, workshops, and seminars specifically arranged for S1 students | i. Voluntary work training; ii. Peer counselors; iii. Environment protection; iv. Antismoking; v. Antidrug; vi. Online addiction; vii. Adoption program; viii. Antibullying; ix. Religious group |
| **II. Basic Information of Tier 1 Program Participation** | |
| Hours | 10-h core program |
| Mode | Mode 4 (20 30-min sessions) |
| Program arrangement | Moral Education (100%, once every week) |
| Rationales of the program arrangement | The topics were implemented in school regular teaching period, which reinforced the systematical arrangement for the development of the Project P.A.T.H.S. |
| Medium of instruction | Chinese |
| Consolidation session | N.A. |
| Introduction session | Yes (Students flew their own paper planes, after they wrote their expectations towards P.A.T.H.S. on them.) |
| Conclusion session | Yes (Students played in a competition with revision of all lessons. Some excellent performances were rewarded.) |
| Supplementary activity | No |
| Other activities | Reward system and competition |
| Student assessment on P.A.T.H.S. program | Yes (sharing) |
| Have joined Experimental Implementation Phase | Yes |
| **III. Information of the Instructors Conducting Tier 1 Program** | |
| No. of instructors who conducted Tier 1 Program | One vice-principal, one teaching assistant, and one school social worker |
| No. of instructors who joined P.A.T.H.S. training workshops | Eight teachers and one school social worker |
| No. of instructors who joined the workshops on "Handling Behavioral and Emotional Problems of Students" | Four teachers and one school social worker |
| No. of instructors who joined the workshops on "Personal Growth and Psychological Well-Being of Teaching Professionals" | Four teachers and one school social worker |
| **IV. Basic Information of Tier 1 Program Implementation** | |
| No. of preparation meetings and content | N.A. |
| No. of instructors who conducted Tier 1 Program in each class | One teacher, one teaching assistant, and one school social worker |
| Person responsible for preparing the teaching materials and the way of preparation | School social worker was mainly responsible for preparing worksheets and teaching materials, as well as implementing the reward system. |

*Table 1 continues*

**TABLE 1 (continued)**

| | |
|---|---|
| Format and frequency of lesson preparation among instructors | Instructors called meetings for preparation before class when necessary, especially when there was special arrangement or fine adjustment during implementation. |
| Format and frequency of experience sharing after class among instructors | Done when necessary for case interventions or other special events. |
| No. of evaluation meetings and content | Comments collected during the class teachers' meetings. |
| Cooperation between the school and social work agency | The teacher took the main role in controlling class discipline, grouping students, delivering aim of games to students, and sharing his own experience; social worker was responsible for leading classroom sharing and discussion, and preparing teaching materials. |
| Role of the teacher in Tier 1 Program implementation | i. Teaching the program; ii. Monitoring classroom discipline; iii. Grouping students |
| Role of the social worker in Tier 1 Program implementation | i. Teaching the program; ii. Preparing teaching materials; iii. Coordinating the program-related matters; iv. Following-up (esp. student counseling); v. Implementing the reward system |

**TABLE 2**
**Consolidated Interpretations on the Administrative Arrangements and Related Issues in the School**

| Aspect | Integrated Findings and Interpretations |
|---|---|
| (a) Support for the program and its rationales | • Strong support from the school contact person, which facilitated the program implementation.<br>• Both teacher and social worker agreed with the project rationales and considered that the rationales matched with students' needs. |
| (b) Perceived program arrangement, and its advantages and disadvantages | • The program was incorporated into a teaching subject of the existing school system.<br>• Advantages: (a) systematic implementation, (b) students' resistance was low as they were required to participate fully in the scheduled timetable, (c) teachers were serious in program delivery.<br>• Disadvantage: Nil. |
| (c) Manpower deployment | • One vice-principal and one social worker delivered the program across all Secondary 1 classes.<br>• One teaching assistant was responsible to facilitate classroom activities and give feedback on student assignment. |
| (d) Facilities for implementation | • Not sufficient video projectors for the implementation in the first semester.<br>• A well-equipped room was provided for the social worker to keep filing.<br>• Inviting school atmosphere and appealing classroom settings. |
| (e) Support within school and from social work agency | • Attributes of the school principal: supportive.<br>• Good collaboration with social work agency and sufficient support during implementation. |
| (f) Impression on the overall administration | • The school contact person was satisfied with the administrative arrangement.<br>• The social worker had positive perceptions of the administration done by the school contact person. |

development. Adopting the program in the school and matching the project's rationales with the positive school ethos are revealed in the narratives of the vice-principal:

> *"Why did we join (the program)? Actually, it matches with our school ethos – proper education without distinction between types of students, whole-person development… We would like to trust them (students), respect them, and believe that they can do many things in appropriate environment… also be optimistic, just like your program does, believe that they can achieve things in the future though they cannot achieve it now, that is we cannot give up… like what are in your program, there are pre-designed PowerPoint, activities, social issues, sharing… very comprehensive and systematic. Therefore, when we heard about your program, we immediately sent application to join it."*

Second, strong administrative support from the principal, in terms of planning the school timetable and arranging manpower deployment for the program implementation a year in advance, was important in program administration. For the program arrangement, the program was incorporated into the existing teaching subject. All respondents perceived this arrangement to be advantageous because there were no labeling effects. Also, students' resistance was low as all of them were required to participate fully in accordance with the school timetable. Moreover, this arrangement allowed systematic implementation and increased the instructors' seriousness in delivering the program. Regarding manpower deployment, there was a clear division of labor in program implementation. The school vice-principal was the contact person of the program and played the main role of delivering the program across all Secondary 1 classes; a teaching assistant helped classroom facilitation and marked students' assignments, while the school social worker was the coordinator of the program. All this administrative planning and arrangement greatly facilitated the program implementation.

Third, high involvement of the school administrative personnel was a crucial factor in facilitating program implementation. The principal revealed that,

> *"…deliberately letting the vice-principal (to teach all four Secondary 1 classes)… because it is hoped that the vice-principal can get acquainted with all Secondary 1 students… because he has a more important position in the students' eyes… Actually, the vice-principal has fewer teaching duties of other subjects, he can have relatively more time to prepare the P.A.T.H.S. lessons and evaluate the teachings with the social worker, which in turn increased the program effectiveness".*

Fourth, as the school had provided a well-equipped room for the social worker to keep filing and related documents, it is obvious that having adequate facilities for the program implementers to carry out the program was necessary. In the interviewed school, there were appealing software and hardware resources that helped to create a hospitable school. It is reflected in the narrative of vice-principal:

> *"We welcomed them (the students) when they entered the school (at Secondary 1 level). It makes them less likely to transfer studying in other schools. Then they develop a sense of school belonging, become obedient to teachers, and finally pay attention in class."*

> *"We claim to be a school of care and love… in this year, we decorate the classrooms as home, like a family…we put sofa, bench, soft toys, the things we like in the classrooms… it develops a sense of belonging. Through play and love, it finally helps students to study and change themselves."*

### Implementation Characteristics in the School

The implementation characteristics in the school are summarized in Table 3. Coteaching was adopted in the school, and a consistent pair (i.e., the vice-principal and school social worker) taught all Secondary 1 classes. They had clear roles in coteaching, and both were cooperative and supportive. The vice-principal regarded themselves as "dream partners" and he highlighted that the involvement of the social worker was indispensable. He also noted that there should be at least two to three instructors teaching a class in order to a have a better control of the classroom discipline. In addition, several teaching strategies were used in the implementation process.

At the beginning of the program implementation, the students were shy and were not used to grouping, but they became familiar with group activities and developed a sense of classroom cohesion. The informants perceived that the students were happy, joyful, and highly involved in the program. Some students also learned to appreciate the instructors. Obviously, different types of students had different responses, e.g., extroverted students were open in sharing themselves in classroom, while introverted students preferred to reveal their feelings on the worksheets. Therefore, students' worksheets were regarded as a vehicle for teacher-student communication. The instructors checked students' assignments, gave feedback, and recognized the students' work, while the students looked forward to receiving the teachers' feedback and recognition. All the students' assignments were kept in the folders, which kept the students informed of their learning progress. Both the vice-principal and social worker perceived having a better relationship with students in the implementation process.

Regarding the difficulties encountered in the implementation, the respondents showed that the students had different attention spans and behavioral problems in the classroom, and they had to guide them with patience and set up a contract of punishment in advance. They also indicated that some teaching units had insufficient or not up-to-date teaching materials, and thus they had to modify them in advance. The vice-principal added that it was meaningful to carry out the program, although much preparation was needed.

### Perceived Program Effectiveness, Program Success, and Overall Impression

As shown in Table 4, the informants perceived that the program was effective as reflected in the high student involvement. The social worker remarked in her narratives that the students could remember what they had learned from the program:

> *"The main teaching focus of the Secondary 1 level this year was interpersonal skills… I used the topics of bonding to teach them. I remembered we had had to choose "the top-ten unpleasant expressions in Hong Kong" in a lesson earlier, then I used it to remind a girl about her attitudes…lastly, she thought for a while and understood."*

In terms of program success, since the program had been implemented for 2 years in the school, the instructors perceived that students had significant improvement. This also let the instructors feel that they were recognized for their effort. They also perceived the program as successful because it was known by the students' parents. One of the reasons was that they had arranged a sharing session for the parents, in which students carried out a fashion show and demonstrated what they had learned in the program.

Regarding the overall impression of the program, the vice-principal perceived that the curriculum was close to daily life, although some examples needed to be updated with reference to social changes. He appreciated the teaching tips provided in the teaching manuals because they were useful to the instructors. On the whole, he revealed that the program was a good channel to build a good teacher-student relationship, which fostered a sense of security and belongingness, as did students' learning and positive changes.

**TABLE 3**
**Consolidated Interpretations on the Implementation Characteristics in the School**

| Aspect | Integrated Findings and Interpretations |
|---|---|
| (a) Teachers' and students' responses in the implementation process | • Both teacher and social worker perceived having a better relationship with students.<br>• The teacher regarded the program implementation as meaningful, although much preparation was needed.<br>• Students were happy, joyful, and highly involved. They appreciated the instructors.<br>• Extroverted students were open in sharing themselves in the classroom, while introverted students preferred to reveal their feelings in the homework. |
| (b) Coteaching | • A consistent pair of instructors (the vice-principal and social worker) taught all Secondary 1 classes.<br>• They had clear role in coteaching, and both were cooperative and supportive. |
| (c) Teaching techniques | • Technique 1: To set up various awards to motivate students' participation.<br>• Technique 2: To motivate and recognize students' according to their qualities.<br>• Technique 3: To check students' assignments and give feedback.<br>• Technique 4: Teachers' personal sharing.<br>• Technique 5: To use symbolic and nonverbal means to control classroom discipline.<br>• Technique 6: To set up a contract of punishment.<br>• Technique 7: To create a warm, relaxing, and trustful classroom atmosphere that encourages learning and sharing.<br>• Technique 8: To update and modify teaching materials according to students' types.<br>• Technique 9: To invite alumni to have personal sharing.<br>• Technique 10: To encourage innovations in learning. |
| (d) Handling students' worksheets | • Checking students' assignments, giving feedback and recognition facilitated teacher-student communication.<br>• Student folders were used. |
| (e) Student assessment in the Tier 1 Program | • No examination. |
| (f) Difficulties encountered and solutions | • Difficulty 1: Students had different attention spans and behavior problems. Solution: Gave guidance with patience and set up a contract of punishment in advance.<br>• Difficulty 2: Teaching materials were insufficient or not up-to-date in some teaching units. Solution: Modifications were needed. |
| (g) Advice for next year's implementation | • To have a person responsible for coordinating teaching and preparation.<br>• To include suitable teachers who have a passion to teach such a kind of positive youth development program.<br>• To cultivate class teachers' responsibility to run the program in the long term.<br>• To collaborate with social work agency. |

### DISCUSSION

Several school administrative and implementation characteristics contributing to the implementation quality of positive youth development programs were identified in this study. The first key factor is positive school ethos and belief in the students' potential. The accounts of the informants showed that the

**TABLE 4**
**Consolidated Interpretations on the Perceived Program Effectiveness, Program Success, and Overall Impression**

| Aspect | Integrated Findings and Interpretations |
|---|---|
| (a) Perceived program effectiveness | • Both teacher and social worker perceived program effectiveness in terms of students' improvement and high involvement. |
| (b) Perceived program success | • Both teacher and social worker perceived program success in terms of students' improvement and parents' recognition. |
| (c) Overall impression | • The teacher had positive views of the teaching manuals.<br>• The teacher regarded the program as a channel to build teacher-student relationships and students' sense of school belonging, which facilitated students' learning and positive changes. |

school had positive ethos and the teachers also shared the views that students had the potential to develop, which totally matched with the philosophy of the positive youth development program that had been adopted. In addition, the teachers not only concurred with the program rationales, they also acted as role models and carried out the messages of positive development in their daily interactions with students. As revealed in the respondents' narratives, they applied what should be taught to their students in the topics of bonding, social competence, and beliefs in the future in daily school life – they trusted and respected their students, took initiative to share with them and care about them, and were optimistic about their growth. All of these aspects definitely facilitated the students' learning and positive growth. These findings are generally consistent with the previous studies that showed that positive school ethos and belief in the students' potential can facilitate the development of students[6,26].

The second factor conducive to successful implementation is an inviting school environment. The phrase "school of care and love" and "a sense of school belonging" was emphasized several times by the informants in the interviews. The accounts of the informants also made it explicit that they had put effort into creating an inviting school environment for their students through decorating the physical settings of the classrooms, as well as through strengthening teacher-student relationships, in hope of fostering the students' sense of belonging to the school. As the students in the interviewed school were categorized as having behavioral problems and low academic ability, the teachers regarded that it was necessary to develop the students' sense of school belonging, which is vital to the cultivation of their obedience to teachers and learning motivation. Actually, promoting a valued identity with the school was instrumental because it was found to be positively related to the students' academic efficacy, motivation, achievement[8,9], liking the school, enjoying class, concern for others, and conflict resolution skills[10]; and negatively related to depressive symptoms, social rejection, school problems[11,13], peer victimization, delinquency, and drug use[12]. The present findings also demonstrated that the school principal and teachers played a vital role in building an intimate, mutual, respectful, accepting, caring, and supportive relationship in the school setting[27,28,29], and modeling the positive interpersonal behaviors[10] for promoting the students' sense of school belonging. The present findings are in line with Benson's[26] statement that a caring school climate is one of the developmental assets that facilitates positive youth development.

The third factor conducive to success in a program implementation is an encouraging classroom environment that has several attributes. First, positive relationships were built to encourage students' disclosure, e.g., to create a relaxing and trustful classroom atmosphere for learning and sharing, instructors took the first step to share their own experiences, to recognize students' disclosure, and to support students through giving feedback. Second, personal growth was encouraged as the instructors would motivate the students' participation and learning according to their qualities. Third, there were rules to control classroom discipline; clear reward and punishment systems to let students understand what behaviors are expected or unexpected in the classroom. Fourth, the instructors often encouraged innovation in the students' learning. The respondents added that students were highly involved and enjoyable in this encouraging classroom environment, and these students' responses could reflect a certain degree of program effectiveness. Hence, these findings help to illustrate how the classroom environment proposed by Trickett and Moos[18] can be created to facilitate the implementation of positive youth development programs in Hong Kong classrooms. These findings would dispute the common myth that curricula-based positive youth development programs cannot be easily implemented in classrooms and can hardly arouse students' interest and involvement in Hong Kong school contexts where academic teaching and learning are highly valued.

The fourth factor is the high involvement of school administrative personnel. In the interviewed school, the principal was supportive in various administrative levels, e.g., adopted the program in the school, integrated the program into school curriculum, and arranged manpower deployment and school facilities. Moreover, the vice-principal assumed a leading role in running the program across all Secondary 1 classes, doing preparation, modifying the modes of activities according to the students' needs, and evaluating the activities with the social worker. The high involvement of the school administrative personnel, with their time and effort paid, in the program implementation not only let the students have the impression that the program was important and valuable, which smoothed the program implementation, it also let the vice-principal get acquainted with all newcomers to the school, build up a good rapport with the students, and let students know to whom they could turn in times of need. In short, the involvement of senior administrative personnel in program administration and execution not only facilitated program success, but also benefited the students' learning and teacher-student relationships. This echoes the school rationale of creating an inviting school environment as mentioned above.

Finally, systematic program arrangement was identified as a factor contributing to success. Incorporation of the positive youth development program into the school formal curriculum with clear goals and directions has often been advocated as one of the crucial factors leading to effective program implementation[14,15]. Also, there were studies that showed that the students were satisfied with and benefited from the developmental program that had been integrated as part of the school curriculum[30]. The present findings explained the fact that this systematic arrangement could raise the seriousness of both teachers and students in their teaching and learning, respectively, and thus the program implementation quality. In addition, the school had a clear manpower deployment with designated roles, in which a consistent pair of instructors taught the program across all Secondary 1 classes throughout the year. This arrangement was planned a year in advance and had justification behind its application. In the administrative aspect, planning well is necessary, and "systematic" and "consistency" are two invaluable elements that enable a sense of stability and security, and cultivate devotion and dedication, which, in turn, helps to facilitate the program implementation and quality.

There are several limitations of the present study. First, as the study was conducted in a school with students having low academic achievement, and it was selected on convenience sampling, the generalization of the findings is questionable. However, it is noted that the beauty of case study or qualitative study is to unravel the uniqueness, rather than to generalize findings to the whole population. Second, as a single interview with three informants only was conducted, the views of other stakeholders, i.e., the teachers who were not involved in the program implementation, the students, and their parents, were not heard. Third, as the data collection and analyses were done by the colleagues and researchers of the project, they might have biases and assumptions of the program effectiveness that affect the credibility of the findings. However, the researchers were conscious of their biases and performed the data analyses in a disciplinary manner to safeguard the reliability of the findings. Fourth, as the face-to-face interview method was used, and one of the interviewers was the cowalker of the interviewed school having an acquainted relationship, the respondents might have a tendency to cooperate with and help issue the program effectiveness. Nonetheless, this was safeguarded as the interview also included another trained interviewer (the third author), and the respondents were encouraged to voice negative views, if any. Finally, the present study was conducted with a school that adopted the 10-h program only; further research based on schools that adopted the 20-h program would be helpful. Despite these limitations, the present findings suggest that the Tier 1 Program of the Project P.A.T.H.S. could be launched effectively in schools with the appropriate administrative and implementation arrangement.

### ACKNOWLEDGMENTS

The preparation for this paper and Project P.A.T.H.S. were financially supported by The Hong Kong Jockey Club Charities Trust.
